# Predictive Data Analytics in Telecare and Telehealth: Systematic Scoping Review

**DOI:** 10.2196/57618

**Published:** 2024-08-07

**Authors:** Euan Anderson, Marilyn Lennon, Kimberley Kavanagh, Natalie Weir, David Kernaghan, Marc Roper, Emma Dunlop, Linda Lapp

**Affiliations:** 1 Department of Computer and Information Sciences University of Strathclyde Glasgow United Kingdom; 2 Department of Mathematics and Statistics University of Strathclyde Glasgow United Kingdom; 3 Strathclyde Institute of Pharmacy and Biomedical Sciences University of Strathclyde Glasgow United Kingdom; 4 Centre for Heart Lung Innovation University of British Columbia Vancouver, BC Canada

**Keywords:** telecare, telehealth, telemedicine, data analytics, predictive models, scoping review, predictive, predict, prediction, predictions, synthesis, review methods, review methodology, search, searches, searching, scoping, home

## Abstract

**Background:**

Telecare and telehealth are important care-at-home services used to support individuals to live more independently at home. Historically, these technologies have reactively responded to issues. However, there has been a recent drive to make better use of the data from these services to facilitate more proactive and predictive care.

**Objective:**

This review seeks to explore the ways in which predictive data analytics techniques have been applied in telecare and telehealth in at-home settings.

**Methods:**

The PRISMA-ScR (Preferred Reporting Items for Systematic Reviews and Meta-Analyses extension for Scoping Reviews) checklist was adhered to alongside Arksey and O’Malley’s methodological framework. English language papers published in MEDLINE, Embase, and Social Science Premium Collection between 2012 and 2022 were considered and results were screened against inclusion or exclusion criteria.

**Results:**

In total, 86 papers were included in this review. The types of analytics featuring in this review can be categorized as anomaly detection (n=21), diagnosis (n=32), prediction (n=22), and activity recognition (n=11). The most common health conditions represented were Parkinson disease (n=12) and cardiovascular conditions (n=11). The main findings include: a lack of use of routinely collected data; a dominance of diagnostic tools; and barriers and opportunities that exist, such as including patient-reported outcomes, for future predictive analytics in telecare and telehealth.

**Conclusions:**

All papers in this review were small-scale pilots and, as such, future research should seek to apply these predictive techniques into larger trials. Additionally, further integration of routinely collected care data and patient-reported outcomes into predictive models in telecare and telehealth offer significant opportunities to improve the analytics being performed and should be explored further. Data sets used must be of suitable size and diversity, ensuring that models are generalizable to a wider population and can be appropriately trained, validated, and tested.

## Introduction

Technologies can play a role in addressing the challenges associated with supporting people to live longer independently at home. Telecare services have existed since the 1970s and are systems designed to support vulnerable individuals living in their homes, enabling them to maintain their autonomy while ensuring protection from any anomalous situations that may arise [1]. Telecare devices have gone through many iterations since their introduction as simple user-triggered alarms and now include, for example, bed occupancy sensors and automatic fall detectors [1]. Today, telecare systems can work as lifestyle monitors, collecting data relating to the individual and their home environment in real time. Telehealth services are used in the management of long-term conditions such as heart disease or diabetes. Users are provided with equipment, such as vital signs monitors, to record blood pressure, heart rate, or blood glucose levels, for example. These data are shared with care providers to allow remote assessment of the well-being of an individual and to intervene if necessary.

Technology-enabled services have been a feature of care at home for a number of years and the demand for these services remains high. In Scotland alone, there are over 129,000 people (2.4% of the total population) who make use of a telecare service or community alarm [2], while an estimated 1.8 million people across the whole of the United Kingdom (2.7% of the total population) use either telecare or telehealth services [3]. In the United States, a total of 2.3 million veterans used telehealth services in 2022, representing more than a third of all veterans receiving care from the Department of Veterans Affairs [4].

Newer telecare and telehealth devices collect increasing amounts of data from a variety of connected sensors and systems. However, most services respond to an anomaly once it has been identified and do not intelligently use the data they receive to identify those at higher risk of an adverse event in order to pre-emptively plan what an individual may require. There are significant benefits to more proactive services, such as a reduction in secondary care use, including ambulance callouts or eventual hospital admissions [5,6].

Recent policy has highlighted a desire to shift telecare and telehealth services toward a more proactive model. The UK Government state—in their plan for Digital Health and Social Care—that anticipatory care promoting prevention through machine learning–facilitated data analysis will be routinely implemented by 2028 [7]. This has similarly been highlighted in a number of other countries including Australia, Canada, and New Zealand [8-10].

This scoping review, therefore, seeks to identify and explore the ways in which predictive data analytics techniques have been applied in the use of community-based telecare and telehealth devices and services in order to identify the current gaps and opportunities that exist for the future use of predictive analytics in telecare and telehealth.

## Methods

This review was conducted and presented in accordance with the PRISMA-ScR (Preferred Reporting Items for Systematic Reviews and Meta-Analyses extension for Scoping Reviews) 2020 checklist [11]. The protocol was informed by the methodological framework proposed by Arksey and O’Malley [12].

### Inclusion or Exclusion Criteria

This review considered any study using quantitative methods relating to the predictive use of data analytics in the fields of telecare and telehealth. Qualitative studies were excluded. The Population, Concept, and Context (PCC) framework was applied. Database searches were conducted in August 2022 and restricted to papers published within 10 years of the initial searches being conducted. Only papers published in the English language were considered.

### Population

Papers focusing on any and all users were included. All populations of users (anyone using a telecare and telehealth device or systems) including both adult and child services were valid for inclusion since the focus of this review was on the methods of analytics being applied, rather than the specific reason for accessing telecare or telehealth.

### Concept

Any telecare or telehealth innovation that gathers or generates data and electronically communicates it for use in an analytical manner was valid for inclusion. This could be “passive” technology, such as sensors and wearables, or “active” technology where data are intentionally entered into a device by a user. Papers investigating devices, which do not directly monitor a health element of an individual, such as an educational app, were excluded. Any data analytics that make inference or predictions from the data they receive were included in this review. This includes diagnosis, classification, and anomaly detection and does not exclusively consider predictions of future events. Additionally, this review only considers telecare and telehealth devices related to a somatic condition, that is, physical condition of the body. Papers focused on mental health and loneliness, for example, were excluded because these conditions may require a significantly different management approach.

### Context

Any paper which had a “care in the community” setting was suitable for inclusion (patient’s own home, assisted living facilities, and sheltered accommodation). In-patient and non–home-based settings were excluded with the exception of papers that focus on technologies clearly designed for at-home use that have thus far only been tested on individuals in an in-patient setting.

### Study Type

All reviews (systematic, literature, and scoping) were excluded as this would cause duplicate data to be reviewed and could lead to bias through overreporting. Any paper outlining an entirely conceptual framework and not detailing on how it would work in practice was excluded. The review also excluded editorials, summaries, and opinion pieces.

### Databases Searched

Databases relevant to health and social care—MEDLINE [OVID], Embase [OVID], and Social Science Premium Collection [ProQuest]—were searched.

### Search Strategy

The following 2 key domains were identified for inclusion in the search strategy: data analytics and telecare or telehealth (see Table 1). Search terms that were deemed most applicable to each database were applied. MeSH (Medical Subject Headings) terms and free-text entries were considered as appropriate. Boolean operators such as “AND,” “OR,” and truncation codes were used to refine and improve searches. A copy of the full search strategy employed while searching the Medline database can be found in Multimedia Appendix 1.

**Table 1:** Synonyms considered during literature searches for review.

Search term domains	Synonyms
Data analytics	Data analytics Big data Health analytics Electronic data capture Data management system Machine learning Data analysis Data mining
Telecare or telehealth	Telecare Telehealth Remote health care services Remote monitoring Telemonitoring Telecommunication Advanced assistive technology

### Study Screening

Results from each database search were imported to EndNote [13] where duplicates were removed. Studies were uploaded to Covidence [14] for screening. Title and abstract screening were completed by 6 reviewers (ML, NW, ED, DK, MR, and LL). Every paper was screened independently by at least 2 researchers, with conflicts resolved through discussion. A third reviewer was consulted when agreement could not be reached.

Full-text versions of the accepted papers were obtained for full-text screening. There were 537 papers considered for full-text screening by the lead author. Of these 537, approximately 15% (n=80) were screened collaboratively by the lead author (EA) and 2 other reviewers (NW and DK). Interrater agreement (all 3 reviewers coming to the same conclusion on inclusion or exclusion) was categorized through the following thresholds: <70%=poor, 70%-79%=fair, 80%-89%=good and ≥90%=excellent [15]. Of the papers that were collaboratively reviewed by all 3 researchers, there was an interrater agreement of 81%. This was a sufficient level of agreement for the remaining full-text papers to be independently screened by the primary author only. A second opinion was sought by the primary researcher during full-text screening when required.

### Data Charting Process

A data extraction table was created in Microsoft Excel by the primary author. The data extraction table was piloted by the primary author for the first 10 papers before a discussion with secondary authors was conducted to ensure the appropriateness of the data being extracted. These discussions helped shape the table further with modifications made so that all relevant pieces of information were extracted. Data extracted related to key study characteristics, data analyzed in the paper, the technology employed, and the analytics techniques used.

### Data Items and Synthesis of Results

Data were collected on paper characteristics (eg, title, authors, year of publication, location of publication, and country of origin) and study characteristics (eg, study design, stage of implementation, study setting, primary or secondary analysis, participant description, duration of study, and dropouts). Data were also captured relating to the technology in use (eg, what the technology is designed to assist with, the technology being employed, and its function), the data used in the analyses (eg, data streams, where the data are sent, and what it is being used for), and the methods of analyses employed (eg, the statistical method of analysis, the actions taken as a result of the analysis and outcome measures). Information on the key findings from each study and any potential limitations with the studies were also collected. A summary of the data extracted for each paper can be found in Multimedia Appendix 2.

## Results

**Figure 1:**
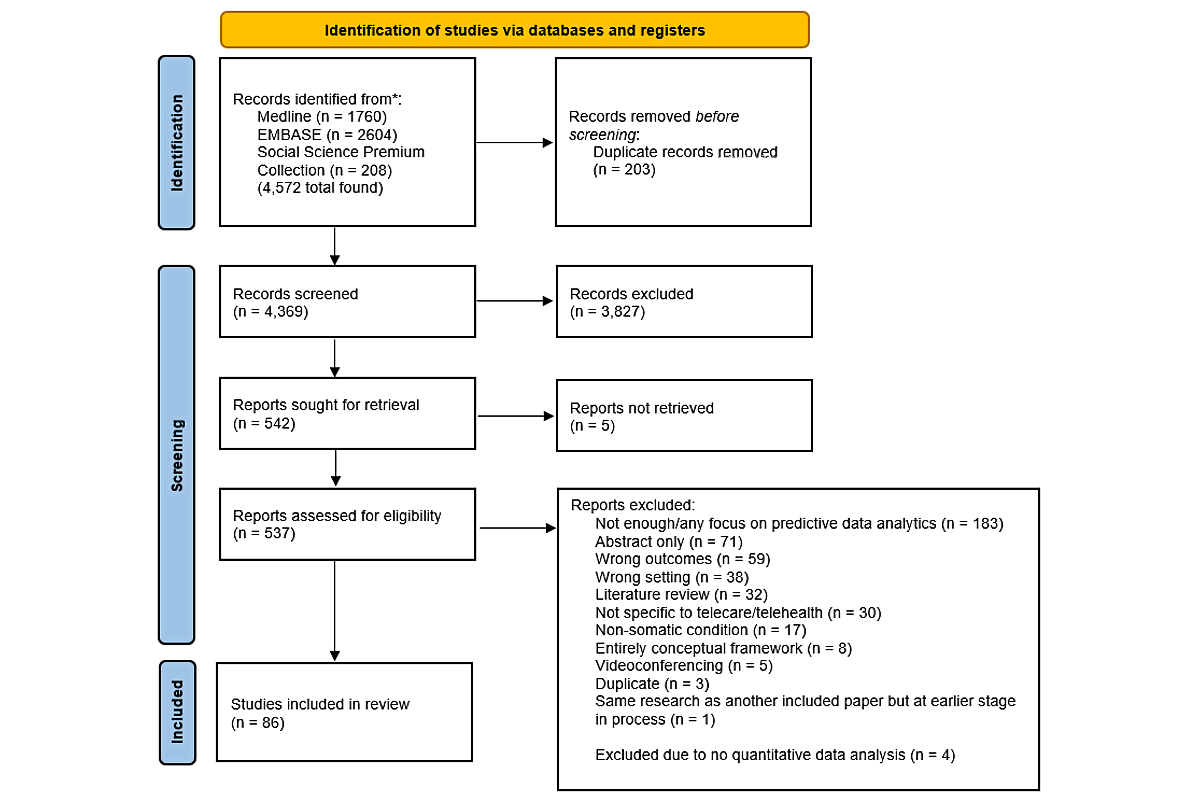
PRISMA (Preferred Reporting Items for Systematic Reviews and Meta-Analyses) flow diagram outlining the full screening process.

A total of 86 published papers were included in the review. A PRISMA (Preferred Reporting Items for Systematic Reviews and Meta-Analyses) flowchart of the full screening process completed for this review can be found in Figure 1. Of the 86 selected papers, approximately one-third of papers (n=28) considered telecare services, with the other two-thirds considering telehealth services (n=58).

The data analytics tasks employed in the studies reviewed (with reference to Banaee et al [16]) can generally be categorized into: anomaly detection (n=21, 24%), prediction (n=22, 26%), and diagnosis and decision-making (n=32, 37%). Additionally, this review identified a fourth data analytics task, which relates to activity recognition systems (n=11, 13%). Table 2 provides a breakdown of the papers, categorized by the type of data analytics task applied.

**Table 2:** Categories of data analytics in included papers.

Type of data analytics applied	Studies, n	References
Diagnosis and decision-making	32	[17-48]
Prediction	22	[49-70]
Anomaly detection	21	[71-91]
Activity recognition	11	[92-102]

The most common areas of focus for overall technology systems were general monitoring systems (n=14, 16%) and activity recognition systems (n=11, 13%). The majority of the included papers focused on the prevention, detection, treatment, or monitoring of a specific health condition (n=53, 62%). Of these, the most commonly studied was Parkinson disease (n=12, 14%), followed by conditions of the cardiovascular (n=11, 13%) and respiratory systems (n=8, 9%). Table 3 lists the number of papers considered by the paper’s focus, split between technology systems and by health condition.

**Table 3:** Focus of papers included in review, grouped by monitoring systems, and by health condition^a^.

		Studies, n	References
**Focus of Paper (Technology System)**			
	General monitoring system	14	[30,53,55,59,71,73-75,80,84,85,88,90,91]
	Activity recognition system	11	[92-102]
	Falls monitoring system	5	[51,70,86,89,100]
**Focus of Paper (Health Condition)**			
	Parkinson disease	12	[20,21,28,32,40,41,43,44,47,48,76,77]
	Cardiovascular system (heart disease, heart failure, atrial fibrillation, cardiovascular disease, blood pressure, and anticoagulation)	11	[23,24,27,46,57,63,67,68,70,82,83]
	Respiratory system (lung transplant, chronic obstructive pulmonary disease, and asthma)	8	[49,56,58,60-62,69,79]
	Sleep apnea	4	[26,29,35,78]
	Diabetes (including prediabetes)	4	[39,64,65,81]
	Poststroke rehab	3	[22,25,31]
	Cognitive assessment or dependence	2	[18,33]
	Weight or diet	2	[36,54]
	Multiple sclerosis	2	[37,45]
	Craniosynostosis	1	[17]
	Gait	1	[19]
	Pressure injuries	1	[34]
	Alzheimer disease	1	[38]
	Typhoid	1	[42]
	Cancer	1	[50]
	Pancreatectomy	1	[52]
	COVID-19	1	[66]
	Knee arthroplasty	1	[72]
	Pain management	1	[87]

^a^Table 3 does not sum to 86 as there are a small number of papers that have more than one area of focus.

Studies featuring primary data sources accounted for just over half of the papers included (n=46, 53%). There were a further 36 papers (42%) that used data originating from secondary sources, such as data gathered over the course of a separate experiment or trial that was then applied to future studies, while 4 papers (5%) used a combination of both primary and secondary sources [27,41,85,99]. There were a total of 3 papers that focused on the predictive analytics of data that has been routinely collected in telehealth practice, while there were no such telecare papers [31,42,68]. Every paper reviewed was either in a pilot or feasibility study or was undergoing proof-of-concept tests.

Table 4 displays the different types of technologies featured in this review. The most common technologies were wearable sensors (n=38, 44%). The majority of the papers (n=68, 79%) used at least 1 type of sensor—be it wearable, environmental or motion or pressure, smartphone, or 3D motion scanners. Other technologies included self-reported symptoms via smartphone apps (n=17, 20%) and vital signs monitoring (n=11, 13%). These technologies do not map neatly onto the data analytics tasks shown in Table 2. For example, wearable sensors feature in papers that consider diagnosis and decision making, anomaly detection, prediction, and activity recognition tasks.

**Table 4:** Technology featured in papers under review^a^.

Technology used	Studies, n
Wearable sensors	38
Patient-reported outcomes via app	17
Environmental/pressure/motion sensors	16
Vital signs monitoring	11
Smartphone sensors	10
3D motion scanners	4
Computer or phone-based testing	2
Virtual glove	2
Virtual knee sleeve	1
Video recording	1
Voice recording	1
Images	1

^a^Table 4 does not sum to 86 as a number of papers featured the usage of more than one technology*.*

Machine learning (ML) techniques were the most commonly applied method of analysis of the data collected in the studies reviewed (n=76, 88%). Table 5 breaks down the ML techniques that have been reported in at least 2 papers in this review, highlighting the variety of different possible methods of analysis. For papers that consider multiple different ML methods, only the technique found to be most accurate has been selected. Other methods of analysis employed in this review were rules-based inference systems (n=4, 5%) and nonmachine learning algorithms (n=3, 3%). The most commonly applied ML methods were decision trees (n=14, 16%), followed by neural networks (n=12, 14%) and support vector machines (n=11, 13%). Additionally, there are a number of papers (n=16, 21%) that consider highly bespoke algorithms, employed in 1 instance only, which do not feature in Table 5.

**Table 5:** Machine learning techniques applied in relevant papers.

Machine learning technique	Studies, n
Decision trees	14
Neural networks	12
Support vector machines	11
Random forests	8
Ensemble (combination of models)	6
Logistic regression	5
Hidden Markov Models	2
k-Nearest neighbors	2

There were 68 papers (79%) in this review that reflected on potential limitations with their studies. Of these, 2 limitations were identified across multiple papers: small sample or study sizes (n=32, 47% of papers reporting limitations) and the issue of bias (n=13, 19% of papers reporting limitations). In total, there were only 2 included papers that considered the calculation of suitable sample sizes for their studies [31,79].

The main limitation identified in the papers reviewed is that a significant number of papers are trained on very small data sets or samples. In total, there were 32 papers that acknowledged this as an issue. The other limitation that was identified a significant number of times was the possibility of the introduction of bias to the models. Bias presents a similar issue to small sample sizes as it can invalidate the findings of a study, as the model is trained on a group that is not representative of the wider population of interest. The types of bias identified in this review can be found in Table 6.

**Table 6:** Sources of bias identified by researchers.

Type of bias	Studies, n	References
Technology trialed on young, healthy individuals	5	[51,71,83,93,100]
Female dominated data set	4	[18,26,39,54]
More complete data received from healthier individuals	1	[24]
Participants almost all White and college educated	1	[44]
Participants all recruited from one church in urban area	1	[59]
Male-dominated data set	1	[67]

## Discussion

Within this review, the data analytics approaches can be categorized, with reference to Banaee et al [16], as: anomaly detection, prediction, and diagnosis or decision-making. Additionally, a fourth analytics category, activity recognition systems, has been identified. Table 2 features a breakdown of the analytics approaches employed in the reviewed papers.

Diagnosis and decision-making systems were the most commonly occurring data analytics task performed in the literature (n=32, 37%), while systems designed to identify anomalous events that have already taken place accounted for 21 reviewed papers (24%). Systems designed to make temporal predictions—identifying anomalies or events before they occur—only accounted for 22 of the papers reviewed (26%). This branch of analytics approaches is of critical importance to researchers and care providers due to the potential health care savings that could be made through the timely and proactive identification and resolution of anomalies before they occur. As such, it is expected that in the future, studies focusing on predicting anomalous events will be more frequently applied in the field of telecare and telehealth. This is supported by recent policy documents highlighting aspirations to move toward more proactive and predictive models of care [7-10].

The final identified branch of data analytics tasks is activity recognition systems (n=11, 13%). These systems typically use a classification model to identify the activity performed (eg, walking and falling), which is very relevant in the field of telecare but found rarely in the literature. A few studies show how such systems could be advanced toward more predictive anomaly detection [92,100] but they do not currently have a feedback loop whereby the recognition of an event taking place leads to an action by the care provider. This is of critical importance if aiming to identify people at risk of an adverse event and take preventative measures and is likely to become more commonly applied in telecare and telehealth moving forward.

### Analytics Focus

This review also highlighted that there has been significantly more research into predictive analytics in telehealth (n=58) compared to telecare (n=28). Telehealth data may be more suitable to the application of predictive analytics because they are often more structured and numerical in nature whereas social care data more frequently rely on unstructured case notes.

Studies which considered a system or technology aimed at a specific disease or condition made up the majority of papers identified, with the most common disease of focus being Parkinson disease [31-42]. The extensive focus on Parkinson disease in research may be attributed, in part, to its features and symptoms and their suitability for being measured by sensors and then modeled by data analytics techniques. For example, slowness of movement, uncontrollable shaking, and gait problems are very common symptoms of Parkinson disease and are all well suited to being captured through wearable sensors. Such remote monitoring or assessment is also useful in diseases like Parkinson disease where clinical features of the disease may be intermittent in the early stages and thus may not be present during a scheduled assessment [103].

### Patient-Reported Outcomes

While patient-reported outcomes (PROs) were one of the more commonly featured tools in this review (n=17, 20%), they are not commonly used in telecare predictive data analytics models (n=3/28 telecare papers, 11%). PROs can provide more nuanced information than solely using clinical indicators which can lead to an underestimation of the impact on a patient in combination with an overestimation of the effectiveness of treatment being provided [104,105]. As such, there is an argument to be made for further use of PROs in predictive data analytics models, especially in the field of telecare.

Including PROs in predictive modeling is challenging as it involves the integrating both objective and subjective data. However, this integration can enhance model results by capturing the true reported experiences and outcomes of patients. Indeed, evidence shows that PRO measurements are of comparable accuracy to many objective clinical measures [106]. Appropriate testing, validation, and re-evaluation of PROs can help improve the quality and consistent collection of data while the move toward standardization of PROs through the use of tools such as the National Institute of Health’s Patient-Reported Outcome Measurement Information System (PROMIS) can enable a rise in data quality levels across the board, facilitating a greater integration of PROs in predictive modeling work [107].

### Use of Routinely Collected Data

Routinely collected data can be defined as data that has not been specifically captured for research purposes. There are only 3 studies featured in this review using data that have been routinely collected in real-world health and care practice, with all of these papers considering telehealth systems [31,42,68]. From a telehealth perspective, a lack of use of routinely collected data makes sense due to these systems focusing on highly specific features that need to be extracted about a given condition or illness. As such, the data considered in these systems tend to originate from bespoke, highly targeted data collection methods.

However, a significant amount of data is being generated by providers of telecare services globally as they deliver care, and the application of data analytics in these real-world data sets needs to be explored further than it has been to date. One key barrier to the analytical use of routinely collected telecare data is that these data are typically siloed in different locations, with systems lacking interoperability. For example, call handling data are frequently maintained in a different system than other social care data, resulting in the outcomes of calls being inaccessible to social care organizations. This has been identified by the Scottish Government as being a key issue preventing the use of data-driven care [108].

Additionally, work must be done to improve other issues surrounding the use of routinely collected data such as patient consent and data governance and security [109]. If researchers, care providers, and any commercial suppliers in control of these rich data sources can collaboratively overcome these identified issues, then a whole new avenue for the use of predictive data analytics will be opened.

### Limitations Within Studies

Limitations noted by researchers were typically specific to the technology employed. These limitations include low quality data being captured [84]; the technology being uncomfortable to wear and with a short battery life [86], and there being a limited number of sensors employed [93]. Limitations related to the analytics techniques included low impact falls being missed by a model [89], large volumes of missing values [61], and a model that struggled to differentiate between an individual sitting and standing [101].

The main limitation identified in this review is that a significant number of papers are trained on very small data sets or samples. In total, there were 32 papers (47% of the total papers reporting limitations) that acknowledged this as an issue. This is a critical problem as having a small sample size could undermine the legitimacy of the findings of the paper—particularly when the outcome of interest is rare. Small sample sizes make it harder to accurately train, validate, and test ML models with the findings less conclusive and less reliable.

To ensure that the strongest evidence base possible sample size calculations should be conducted prior to the study, however only two of the papers featured in this review reported prior sample size estimation [31,79]. This may be attributed to the pragmatic nature of recruitment, where it is difficult to recruit sufficient numbers of individuals with a certain condition, but it remains critical for ensuring the validity of the findings.

The other limitation that was identified a significant number of times was the possibility of the introduction of bias to the models, as can be seen in Table 6. Bias could invalidate study findings as the model is trained on a group that is not representative (eg, gender, age) of the target population meaning that its performance may not translate in reality. In the field of telecare and telehealth, it is critical that data sets consider individuals of appropriate age—generally elderly—and that disease-specific systems have been trialed on individuals with the illness or condition of interest. For example, a study using young, healthy volunteers to classify falls—and other activities—requires participants to simulate falls [100]. This may have an impact on the accuracy of the model, and a data set featuring genuine falls captured by elderly individuals would be significantly more appropriate. The key sources of bias identified in this review are the use of exclusively young, healthy adults to trial technologies that are designed for an older population and data sets, which are dominated by women.

### Limitations of This Review

The quality of the studies selected for inclusion in this review was not assessed using any official appraisal tool. This is typical of a scoping review, which seeks to synthesize the available literature rather than provide a systematic analysis; however, this means that the quality of the papers featured in this review cannot be guaranteed. Another limitation of this review is that it may have missed commercially developed data analytics tools that have been implemented in practice, as these may not necessarily be documented in research literature. Finally, only papers available in the English language were considered, which may preclude a number of relevant papers from this review.

### Conclusions

Predictive data analytics have been widely used in the field of telecare and telehealth but all of the studies featured in this review are still small-scale pilot studies and must be extended to larger trials. Additionally, opportunities for predictive analytics revolving around routinely collected data and PROs should be explored further. Using larger and more diverse “real world” data will enable models to be built that have less bias, can predict more accurately, and could be adapted more widely within other telecare or telehealth settings. Ultimately, appropriate consideration of these factors could lead us to more predictive and preventative data driven models of telecare and telehealth.

## Appendix

Multimedia Appendix 1 Example literature search strategy employed in Medline.

Multimedia Appendix 2 A summary of the data extracted for each paper included in this review.

Multimedia Appendix 3 PRISMA-ScR checklist.

## Abbreviations

MeSH: Medical Subject Headings
ML: machine learning
PCC: Population, Concept, and Context
PRO: patient-reported outcome
PRISMA: Preferred Reporting Items for Systematic Reviews and Meta-Analyses
PRISMA-ScR: Preferred Reporting Items for Systematic Reviews and Meta-Analyses extension for Scoping Reviews
PROMIS: Patient-Reported Outcome Measurement Information System
