# Supplementary material for: Predictive Data Analytics in Telecare and Telehealth: Systematic Scoping Review
Source: Online J Public Health Inform. 2024 Aug 7;16:e57618. doi: 10.2196/57618 (PMC11339581; doi:10.2196/57618)
Supplement: Multimedia Appendix 1 [file ojphi_v16i1e57618_app1.docx]

Multimedia Appendix 1

Below, in Figure 1, is a copy of the search strategy employed during the literature search of the Medline database.


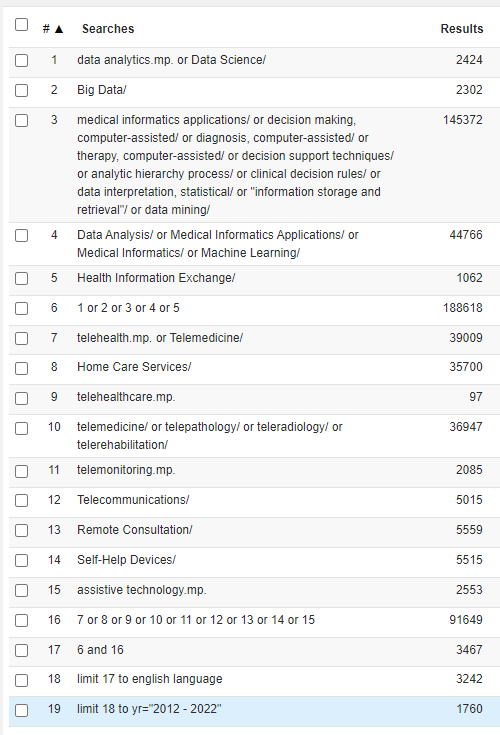


Figure 1 - Search strategy used whilst searching Medline.
