# Supplementary material for: Predictive Data Analytics in Telecare and Telehealth: Systematic Scoping Review
Source: Online J Public Health Inform. 2024 Aug 7;16:e57618. doi: 10.2196/57618 (PMC11339581; doi:10.2196/57618)
Supplement: Multimedia Appendix 2 [file ojphi_v16i1e57618_app2.docx]

Multimedia Appendix 3 – Summary of the data extracted for each paper included in review.

| ***Diagnosis and Decision Making*** | | | | | | | | | | |
| --- | --- | --- | --- | --- | --- | --- | --- | --- | --- | --- |
| ***Title*** | ***Authors*** | ***Year*** | ***Telecare or Telehealth*** | ***Primary or Secondary Data*** | ***Focus of Technology*** | ***Function of Technology*** | ***Technology being used*** | ***Data streams*** | ***Statistical method of analysis*** | ***Study Findings*** |
|  |  |  |  |  |  |  |  |  |  |  |
| Image processing and machine learning for telehealth craniosynostosis screening in newborns | Bookland, M. J.; Ahn, E. S.; Stoltz, P.; Martin, J. E. | 2021 | Telehealth | Secondary | Craniosynostosis | To identify the presence of various types of craniosynotosis in newborns | Images of babies' craniums of suitable quality with midface visibility and visibility of the cranial equator | Values extracted from images: Cephalic index, cranial vault asymmetry index, anterior-middle width ratio, anterior-posterior width ratio and left-right height ratio | Linear discriminant analysis (LDA) ML model | Accuracy of model at detecting normal head shape = 91.7%. Accuracy of model at detecting defects from images higher (range: 96.7% - 100%) |
| A machine learning approach for semi-automatic assessment of IADL dependence in older adults with wearable sensors | Garcia-Moreno, F. M.; Bermudez-Edo, M.; Rodriguez-Garcia, E.; Perez-Marmol, J. M.; Garrido, J. L.; Rodriguez-Fortiz, M. J. | 2022 | Telecare | Primary | Dependence | To automatically assess and predict the dependence of an individual while they perform an instrumental activity of daily living | Empatica E4 Wristband and Samsung Gear S3 Smartwatch | Wristband: Accelerometer (x, y and z axis), Heart rate, Electrodermal activity, Infrared Thermopile. Smartwatch: Gyroscope (x, y and z axis) | k-Nearest neighbour (kNN), random forest and support vector machines (SVM) ML models | kNN algorithm, validated with a 5-fold stratified cross-validation technique achieved an F1 score of 97%, with similar accuracy. Model has only 10 features from initial 85 obtained. |
| A comparison of machine learning classifiers for smartphone-based gait analysis | Altilio, R.; Rossetti, A.; Fang, Q.; Gu, X.; Panella, M. | 2021 | Telecare | Primary | Gait | Record users stride data to allow for machine learning classification of gait | Smartphone app and in-built sensors | Accelerometer and gyroscope data | LDA, quadratic discriminant analysis (QDA), kNN, naïve Bayes, SVM, neuro-fuzzy classifier, classification and regression tree (CART), probabilistic neural network (PNN) and fuzzy inference system | Up to 100% accuracy of classification of gait movement. Probabilistic Neural Network model offered best performance (average accuracy = 91.1%) |
| Sparse Adaptive Graph Convolutional Network for Leg Agility Assessment in Parkinson's Disease | Guo, R.; Shao, X.; Zhang, C.; Qian, X. | 2020 | Telehealth | Secondary | Parkinson's | To provide an automatic assessment of the leg agility task in the MDS-UPDRS | OpenPose to sequence 25 joints of the human body in each frame | 2D coordinates for each joint extracted | Sparse adaptive graph convolutional network | Sample-independent model offered an overall classification accuracy of 70.34% and an acceptable accuracy of 98.97% |
| Estimation of Parkinson's disease severity using speech features and extreme gradient boosting | Tunc, H. C.; Sakar, C. O.; Apaydin, H.; Serbes, G.; Gunduz, A.; Tutuncu, M.; Gurgen, F. | 2020 | Telehealth | Secondary | Parkinson's | To estimate the severity of an individual's Parkinson's Disease | Voice recordings of patient's voice | Demographic and patient info (e.g. age, gender, time since diagnosis). Features extracted from voice recording include: vocal fold excitation ratio, harmonics to noise ratio, mel-frequency cepstral coefficient, recurrence period density entropy, detrended fluctuation analysis, pitched period entropy, wavelet transform, glottis quotient, glottal to noise excitation, empirical mode decomposition and TQWT-based features | Decision tree extreme gradient boosting algorithm | Age and years since diagnosis are effective covariates with speech features. Best ML model offered lowest absolute mean error of 3.87 with Spearman correlation = 0.46 |
| Development and Clinical Evaluation of a Web-Based Upper Limb Home Rehabilitation System Using a Smartwatch and Machine Learning Model for Chronic Stroke Survivors: Prospective Comparative Study | Chae, S. H.; Kim, Y.; Lee, K. S.; Park, H. S. | 2020 | Telehealth | Primary | Post-stroke rehab | A home-based rehabilitation system that can recognise the type and frequency of arm rehabilitation exercises being completed | Commercial smartwatch (LG, style W270) and personal smartphone with custom built app | Inertial measurement unit sensor within smartwatch- produced: three-axis accelerometer and gyroscope data | Convolutional Neural Network trained ML algorithm | The ML model trained with accelerometer and gyroscope data offered the best performance at detecting home exercises most accurately (trained on individual exercise data, not total data): accuracy of 99.9%. In rehabilitation group, statistically significant improvement in WMFT and shoulder ROM. No difference in FMA-UE or grip power |
| A Wearable Electrocardiogram Telemonitoring System for Atrial Fibrillation Detection | Shao, M.; Zhou, Z.; Bin, G.; Bai, Y.; Wu, S. | 2020 | Telehealth | Secondary | Atrial fibrillation | To classify an ECG reading into normal, atrial fibrillation, noisy or other arrhythmia | ECG patch connected to Android smartphone app for data transfer | 31 features extracted from ECG, including: AF Evidence, Shannon Entropy, Kolmogrov-Smirnov Test, Morphology features, RR interval features (median and Index of Arrhythmia) and Features of Noisy class (similarity index of QRS, signal quality index, Q-R smoothness index | Decision tree ensemble CatBoost machine learning classification model | CatBoost model outperforms other models using same dataset. F1 for test and training set both = 0.92. Sensititvity = 99.61%, Specificity = 99.64% and Accuracy = 99.62%. |
| A Machine Learning Approach to Classifying Self-Reported Health Status in a Cohort of Patients With Heart Disease Using Activity Tracker Data | Meng, Y.; Speier, W.; Shufelt, C.; Joung, S.; J, E. Van Eyk; Bairey Merz, C. N.; Lopez, M.; Spiegel, B.; Arnold, C. W. | 2020 | Telehealth | Primary | Heart disease | Classify and determine patient-reported outcomes from passive activity tracking data | Fitbit Charge 2 | Fitbit output (steps, total distance, very active/moderate active/light active distance, very active/fairly active/light active/sedentary active minutes, calories, floor, calories BMR, marginal calories, resting heart rate. PRO questionnaire completed weekly for comparison | Hidden Markov ML Model and Random Forest model | AUC values indicate time-dependent HMM performs better at classifying PROs. AUCs related to physical health PROs higher than for mental health PROs, indicating model better suited to classifying physical health. Indication that PROs cannot be predicted alone from activity tracking data |
| Detection of the Intention to Grasp During Reaching in Stroke Using Inertial Sensing | van Ommeren, A. L.; Sawaryn, B.; Prange-Lasonder, G. B.; Buurke, J. H.; Rietman, J. S.; Veltink, P. H. | 2019 | Telehealth | Primary | Post-stroke rehab | To detect intention to grasp during reach and grasp movements for stroke victims | SEM Glove with force sensors and inertial measurement system | Features extracted from inertial sensors: norm angular velocity vector for middlefinger, index finger, forearm and thumb. Also relative angular velocity (x, y and z axis) for middle finger, index finger, forearm and thumb. Also some additional relative angular velocities in one axis only | SVM model | Best model only uses mean and sd of relative angular velocities of the middle finger (all axes). Single-user classification yielded accuracy of 96.8% and multi-user classification yielded accuracy of 83.3%. Intent to grasp could be detected between 300-750ms earlier than using glove in standard set-up |
| Design and Evaluation of a Non-Contact Bed-Mounted Sensing Device for Automated In-Home Detection of Obstructive Sleep Apnea: A Pilot Study | Mosquera-Lopez, C.; Leitschuh, J.; Condon, J.; Hagen, C. C.; Rajhbeharrysingh, U.; Hanks, C.; Jacobs, P. G. | 2019 | Telehealth | Primary | Sleep apnea | To assess and classify severity of obstructive sleep apnea (OSA) | in-home sleep apnea test (HSAT) sensor system and in-lab polysomnography (PSG). Load cells (LCs) used during every test | Features extracted through Fast Fourier Transform to calculate either skewness or kurtosis of six frequency sub-bands (0.06-0.08Hz, 0.36-0.38, 0.96-0.98, 1.18-1.2, 1.4-1.42 and 1.68-1.7) | Two stage ML model: 1st = decision tree, 2nd = linear regression model | System capable of identifying patients with OSA with an accuracy of 82.9% (sensitivity = 88.9%, specificity = 76.5%). Severity classification accuracy = 74.3% |
| Bimodal classification algorithm for atrial fibrillation detection from m-health ECG recordings | Kruger, G. H.; Latchamsetty, R.; Langhals, N. B.; Yokokawa, M.; Chugh, A.; Morady, F.; Oral, H.; Berenfeld, O. | 2019 | Telehealth | Primary and secondary | Atrial fibrillation | To categorise ECG data into sinus rhythm and AF | Hand-held ECG research device | Factors extracted from the ECG data: time-domain, frequency-domain and physiological features | Bimodal classification algorithm | Performance of optimal classification boundary for both data sets combined: Positive predictive value = 95%, sensitivity = 100% and specificity = 93% |
| Multi-Source Ensemble Learning for the Remote Prediction of Parkinson's Disease in the Presence of Source-Wise Missing Data | Prince, J.; Andreotti, F.; De Vos, M. | 2019 | Telehealth | Secondary | Parkinson's | To allow for the prediction of Parkinson's disease in the presence of missing data (only 8.8% of individuals in dataset had full data) | Smartphone sensor | Accelerometer and gyroscope data. Time domain features extracted (including no. of steps, cadence, skewness, ave. step length, gait velocity, entropy rate etc), frequency domain features extracted (including harmonic ratio, ave. power, signal noise ratio, peak frequency, bandwidth etc), statistical features extracted (including kurtosis, standard deviation etc). Total features extracted for: tapping = 97, walking = 180, voice = 326 and memory = 3 | Four classifiers used: logistic regression, random forest, deep neural networks and convolutonal neural networks | Model that offers best performance in presence of missing values was an ensemble of LR, RF, DNN and CNN which gave a classification accuracy of 82%. |
| Evaluation of Machine-Learning Approaches to Estimate Sleep Apnea Severity From At-Home Oximetry Recordings | Gutierrez-Tobal, G. C.; Alvarez, D.; Crespo, A.; Del Campo, F.; Hornero, R. | 2019 | Telehealth | Primary | Sleep apnea | To allow for the estimation of the severity of sleep apnea | Portable oximeter (Nonin WristOx2 3150) | Single-channel blood oxygen saturation (SpO2) feature extraction: 1st to 4th order moments, mean, sd, skewness, kurtosis, central tendency measure, Lempel-Ziv complexity, sample entropy, 8 features extracted from power spectral density, median frequency and spectral entropy. Oxygen desaturation index also used | ML models: Linear discriminant analysis, 1-vs-all logistic regression, Bayesian multilayer perceptron and AdaBoost | AdaBoost model with linear discriminants as base classifiers offered best performance: Achieved Cohen's Kappa of 0.479 (measure of agreement between actual SAHS severity levels and predicted ones while avoiding the effect of agreement due to chance) and accuracies of 92.9%, 87.4% and 78.7% in binary classifications for increasing severity. Median frequency is only variable not significant and therefore removed from model |
| Mining telemonitored physiological data and patient-reported outcomes of congestive heart failure patients | Mlakar, M.; Puddu, P. E.; Somrak, M.; Bonfiglio, S.; Lustrek, M.; Chiron; HeartMan research, projects | 2018 | Telehealth | Secondary | Monitoring system | To assess the effect of physiological and ambient parameters on the patient-reported outcomes (PRO) | Wearable ECG, activity sensors, body temperature and sweat sensors. Plus daily PRO reporting | Body humidity, body temp, PR interval, QRS duration, T-wave amplitude, QT interval, RR interval, R-wave amplitude, duration of physical activity, energy expenditure, systolic and diastolic blood pressure, oxygen saturation, weight, ambient temp and humidity and PRO indicating how individual feels. Plus various created features such as averages, s.d.s, ratios etc | Random forest, naïve bayes, decision tree and sequential minimal optimization ML models | Best feature subsets = No_sparse_features_0.17_kNN. Classification accuracy for this subset: RF = 86.78% (AUC = 0.79) and Decision tree = 86.1% |
| A low cost virtual reality system for home based rehabilitation of the arm following stroke: a randomised controlled feasibility trial | Standen, P. J.; Threapleton, K.; Richardson, A.; Connell, L.; Brown, D. J.; Battersby, S.; Platts, F.; Burton, A. | 2017 | Telehealth | Primary | Post-stroke rehab | Rehabilitation of arm post-stroke | Virtual glove with infra-red light emitting diode on each finger to track movement | Position of each finger recorded to allow for interactive rehabiliation game | Motion tracking | Sample size not big enough, 38 required per group. Significant change from baseline in intervention group for midpoint Wolf Grip test and two subscales of final Motor Activity Log |
| Detection of Motor Impairment in Parkinson's Disease Via Mobile Touchscreen Typing | Arroyo-Gallego, T.; Ledesma-Carbayo, M. J.; Sanchez-Ferro, A.; Butterworth, I.; Mendoza, C. S.; Matarazzo, M.; Montero, P.; Lopez-Blanco, R.; Puertas-Martin, V.; Trincado, R.; Giancardo, L. | 2017 | Telehealth | Primary | Parkinson's | To detect motor impairment in individuals with Parkinson's disease | Alternating finger-tapping test completed on computer | Features extracted: Skewness and kurtosis, covariance and associated standard deviations. Also sum of the absolute values of the covariance vector elements | Logistic regression, Linear SVM and AdaBoost with different feature selection methods: L1 (lasso), L2 (ridge) and gini impurity | Best univariate model (sum of the absolute values of the covariance vector elements) offers AUC = 0.91, sensitivity = specificity = 0.81. Best multivariate model (Linear SVM with L1 feature selection) offers AUC = 0.88, sensitivity = 0.73 and specificity = 0.84. These are an improvement on reference test: AUC = 0.85, sensitivity = 0.75 and specificity = 0.78 |
| Automated Cognitive Health Assessment From Smart Home-Based Behavior Data | Dawadi, P. N.; Cook, D. J.; Schmitter-Edgecombe, M. | 2016 | Telecare | Primary | Cognitive assessment | To predict the cognitive and mobility health of an individual from their performace of ADLs | Combination motion/light sensors on smart home ceiling, combination door/temperature sensor on cabinets and doors | Features extracted: Mobility (for all activities): total distance travelled and total sensor events. ADLs: sleep duration, sleep sensor events, bed toilet transition duration, cook duration, eat duration, relax duration, personal hygiene duration and leave home duration | SVM, Random forest ML models and linear regression | SVR model offers best performance out of all ML models tested (r = 0.72, RMSE = 14.90 for RBANS & r = 0.45, RMSE = 5.87). Statistically significant results |
| Measuring Repositioning in Home Care for Pressure Injury Prevention and Management | Gabison, S.; Pupic, N.; Evans, G.; Dolatabadi, E.; Fernie, G.; Dutta, T. | 2022 | Telecare | Primary | Pressure injury | To determine the position of an individual as they sleep in bed (to help avoid pressure injury) | 4 single axis load cells (each comprising 4 strain gauges) placed under each bed leg | Features extracted from load cells: mean centre of mass (CoM) paralled to width/length of bed, the ratio of width/length CoMs, s.d. of both CoMs, ratio of s.d.s of CoMs, CoM angle during inhalation phase (plus s.d.), RMS of both components during inhale and exhale phase, ratio of these RMS values, RMS of load signal. Video feed used to provide ground truth labelling of sleeping positions to assess accuracy of models | Eight ML models (w/ incremental learning) considered: AdaBoost, gradient boosting, light gradient boosting, logistic regression, two multilayer perceptrons, SVM and XGBoost | Best performing model for classifying sleeping position: XGBoost classifier with an accuracy of 98.1% and an F1 score of 0.982. Inter-rater reliability analysis score of 0.935 indicates almost perfect agreement between ground truth positions and model |
| R-R interval-based sleep apnea screening by a recurrent neural network in a large clinical polysomnography dataset | Iwasaki, A.; Fujiwara, K.; Nakayama, C.; Sumi, Y.; Kano, M.; Nagamoto, T.; Kadotani, H. | 2022 | Telehealth | Secondary | Sleep apnea | To detect the presence of sleep apnea syndrome (SAS) | PSG system: Alice6LDe or Alice 5 | EEG, ECG, electromyography, SpO2, chest and abdominal wall movements, nasal airflow and a thermistor | Long Short-term memory recurrent neural network | Method able to classify severe SAS with AUC of 0.92, sensitivity of 0.8 and specificity of 0.84. Method able to classify moderate/severe SAS with AUC of 0.89, sensitivity of 0.75 and specificity of 0.87 |
| e-BMI: A gait based smart remote BMI monitoring framework implementing edge computing and incremental machine learning | Adhikary, S.; Ghosh, A. | 2022 | Telecare | Primary | Weight | To categorise individuals into BMI weight classifications | Smartphone sensors | Tri-axial accelerometer and gyroscope data | Incremental machine learning through Stochastic Gradient Descent algorithm. Edge computing also employed to improve performance | Best performing edge computing based machine learning model offered accuracy of 98.6%. Traditional machine learning offered accuracy of 94.8%. |
| Smartphone-and Smartwatch-Based Remote Characterisation of Ambulation in Multiple Sclerosis during the Two-Minute Walk Test | Creagh, A. P.; Simillion, C.; Bourke, A. K.; Scotland, A.; Lipsmeier, F.; Bernasconi, C.; Van Beek, J.; Baker, M.; Gossens, C.; Lindemann, M.; De Vos, M. | 2021 | Telehealth | Primary | Multiple sclerosis | To characterise the ambulation of individuals with multiple sclerosis | Samsung Galaxy S7 and Motorola 360 Sport smartwatch | Tri-axial accelerometer and gyroscope, step count. Energy features: energy, entropy and Teager-Kaiser Energy Operator (TKEO). Statistical features: mean, s.d., skewness, kurtosis, zero-crossing rate and auto-correlation coefficients | Logistic regression, SVM and random forest ML models | Best performing model was SVM with a radial basis function from smartphone data only: Moderate - Acc = 82.2%, sens = 80.1%, spec = 87.2%, F1 = 84.3%. Mild - Acc = 82.3%, sens = 71.6%, spec = 87%, F1 = 75.1% |
| Smart Home-Based Prediction of Multidomain Symptoms Related to Alzheimer's Disease | Alberdi, A.; Weakley, A.; Schmitter-Edgecombe, M.; Cook, D. J.; Aztiria, A.; Basarab, A.; Barrenechea, M. | 2018 | Telehealth | Primary | Alzheimer's | To detect symptoms found to be imaired in Alzheimer sufferers | Various smart home sensors | Day-level features: time spent cooking, eating, relaxing, performing personal hygiene, night time toilet activities, time out of home sleep duration and frequency, total number of activated sensors, total distance covered while walking inside home, complexity of daily routine, number of total and non-repeated activities, max and min inactivity time, day length and similarity with previous day | Random forest, SVM, AdaBoost and Multilayer Perceptron (MLP). To overcome bias, SMOTE-Boost and wRACOG considered | Best performing model at detecting a reliable change in arm curl scores: RF, F-score of 0.77 and sensitivity of 0.92 |
| A remote healthcare monitoring framework for diabetes prediction using machine learning | Ramesh, J.; Aburukba, R.; Sagahyroon, A. | 2021 | Telehealth | Secondary | Diabetes | To facilitate the prediction of diabetes | Samsung Note 8, Huawei GT 2 smart-watch, iHealth blood pressure monitor, iHealth glucometer and iHealth pulse oximeter | Number of pregnancies, glucose level, diastolic blood pressure, skin fold thickness, body mass index, serum insulin level, age and a diabetes hereditary factor pedigree function | kNN, logistic regression, Gaussian Naïve-Bayes and SVM with radial basis kernel (SVM-RBF) | Best performing model at predicting diabetes: SVM-RBF with 83.2% accuracy, 87.2% sensitivity and 79% specificity |
| Telemonitoring Parkinson's disease using machine learning by combining tremor and voice analysis | Sajal, M. S. R.; Ehsan, M. T.; Vaidyanathan, R.; Wang, S.; Aziz, T.; Mamun, K. A. A. | 2020 | Telehealth | Secondary | Parkinson's | To detect the presence of Parkinson's | Smartphone | Tri-axial accelerometer data and vocal recording of sustained vowel sound | kNN, SVM and Naïve Bayes ML algorithms | For voice data and tremor data, knn performs best with accuracy of 98.3% and 98.5% respectively. Ensemble model was able to achieve an average accuracy of 99.8% |
| Building a Machine-Learning Framework to Remotely Assess Parkinson's Disease Using Smartphones | Chen, O. Y.; Lipsmeier, F.; Phan, H.; Prince, J.; Taylor, K. I.; Gossens, C.; Lindemann, M.; Vos, M. D. | 2020 | Telehealth | Primary and secondary | Parkinson's | To discriminate between healthy individuals and people with Parkinson's and to assess severity of disease | Galaxy S3 Mini | Balance, dexterity, gait, rest tremor, postural tremor and voice (not specified exactly what features extracted from these) | Elastic-net regularised regression model | Disease Classification: Accuracy = 0.972, specificity = 0.971, sensitivity = 0.973. Disease severity: Significant correlation between predicted scores and clinician determined scores (r = 0.72, p < 0.0001) |
| Development of an Intelligent Mobile Health Monitoring System for the Health Surveillance System in Indonesia | Djawad, Y. A.; Suhaeb, S.; Ridwansyah; Jaya, H.; Fathahillah; Saharuddin | 2021 | Telehealth | Secondary | Typhoid | Predict whether user is suffering from typhoid, cough or diarrhoea | Online mhealth app with predictive model | Binary yes:no response to 9 symptoms related to illnesses | SVM model | Best model (RBF w/ D = 2, gamma = 0.0 or 0.1) produced correct prediction of 80% with a relative absolute error = 60.74% and ROC area = 0.903 |
| Machine learning-based motor assessment of Parkinson's disease using postural sway, gait and lifestyle features on crowdsourced smartphone data | Abujrida, H.; Agu, E.; Pahlavan, K. | 2020 | Telehealth | Secondary | Parkinson's | To assess the motor abilities of Parkinson's sufferers and discern between Parkinson's sufferers and healthy controls | Smartphone sensor | Accelerometer and gyroscope data. Time domain features extracted (including no. of steps, cadence, skewness, ave. step length, gait velocity, entropy rate etc), frequency domain features extracted (including harmonic ratio, ave. power, signal noise ratio, peak frequency, bandwidth etc), statistical features extracted (including kurtosis, standard deviation etc), lifestyle features including exercise information, smoker status, age, gender also included | Multiple ML models: random forest, bagged trees, cubic SVM, weighted KNN, logistic regression, fine tree, quadratic discriminant and linear discriminant | Best performing models for classifying motor severities: Walking balance = Random forest (precision = 92%, accuracy = 93%, AUC = 0.97), Shaking tremor = Bagged trees (p = 95%, a = 95%, AUC = 0.92), Freeze of gait = Bagged trees (p = 96%, a = 98%, AUC = 0.98). Best model for discerning between Parkinson's and healthy controls = Random forest (p = 94%, a = 95%, AUC = 0.99) |
| Using smartphones and machine learning to quantify Parkinson disease severity the mobile Parkinson disease score | Zhan, A.; Mohan, S.; Tarolli, C.; Schneider, R. B.; Adams, J. L.; Sharma, S.; Elson, M. J.; Spear, K. L.; Glidden, A. M.; Little, M. A.; Terzis, A.; Ray Dorsey, E.; Saria, S. | 2018 | Telehealth | Primary | Parkinson's | To provide a Parkinson's disease severity score from smartphone assessments | HopkinsPD app on Android phones | Total of 435 unique features extracted from 5 tasks. | Rank-based ML algorithm: disease severity score learning | Model correlated well with MDS-UPDRS total (r = 0.81, p < 0.001) |
| Remote monitoring in the home validates clinical gait measures for multiple sclerosis | Supratak, A.; Datta, G.; Gafson, A. R.; Nicholas, R.; Guo, Y.; Matthews, P. M. | 2018 | Telehealth | Primary | Multiple sclerosis | To remotely assess walking speed and how predictive the 25 foot walk test is for real-life walking | AX3-Axitivity accelerometer | Distance walked and spped of walk. Plus tri-axial accelerometry data. Features extracted: mean, variance, max, min, range, max-mean, step amplitude, RMS and step duration) | SVM model | Model built using generalised model from healthy volunteers offered poor prediction power. However, personalised models based on data obtained during calibration stage offered much better performance (R-value = 0.98). From personalised models, can prove that the 25 foot walk is highlight predictive of maximum sustained gait speed at home (R-value = 0.89) |
| A Web Based Cardiovascular Disease Detection System | Alshraideh, H.; Otoom, M.; Al-Araida, A.; Bawaneh, H.; Bravo, J. | 2015 | Telehealth | Secondary | Cardiovascular disease | To classify ECG readings to detect cardiovascular disease | ECG sensor | Indicators extracted from ECG for analysis include: heart rate, QRS interval, P-R interval, P amplitude, Q amplitude, T amplitude, R amplitude, Q width, R width, depression. Other demographic info: age, gender, weight and height | J48 Machine Learning Algorithm (Decision Tree) - BEST, Jrip (rule-based classifier), Kstar (nearest neighbour classifier), Artificial Neural Network and Support Machine Vector | J48 algorithm offers best performance at classifying cardiovascular disease with accuracy = 98.29% |
| Machine learning classification of medication adherence in patients with movement disorders using non-wearable sensors | Tucker, C. S.; Behoora, I.; Nembhard, H. B.; Lewis, M.; Sterling, N. W.; Huang, X. | 2015 | Telehealth | Primary | Parkinson's | To determine whether an individual suffering with Parkinson's is on or off their medication | Microsoft Kinect multimodal sensor | Kinect sensor tracks 20 joints in body. 1890 gait related features extracted from tracking of 20 joints: 60 3D position coordinates, 60 velocity, 60 acceleration and 1710 ratios between these values | Naïve Bayes, IBK classification, SVM, C4.5 Decision Tree | Generalised model (C4.5 Decision Tree) built with multiple individuals' data offered an accuracy of 77.9% (model also had a precision of 1 - i.e. identified all instances of a person being off medication). C4.5 model trained on personal data was able to achieve accuracies up to 100% |
| Analyzing Activity Behavior and Movement in a Naturalistic Environment Using Smart Home Techniques | Cook, D. J.; Schmitter-Edgecombe, M.; Dawadi, P. | 2015 | Telecare | Primary | Parkinson's | To identify activities performed by individuals and see if there is a classifiable difference between healthy individuals and those with Parkinson's | Motion sensors, light sensors, door sensors, temperature sensors and vibration sensors in smart home plus wearable sensor recording accelerometer, gyroscope magnetometer data | Key features extracted for analysis - Environmental sensors: duration of activity, number of sensors activated etc. Wearable sensor (these data streams present for each sensor feature obtained): max, min, sum, mean, median, SD, absolute deviation, coefficient of variation, skewness, kurtosis, signal energy, power, SMA and correlation. Also binary values for success of tasks, age and activity number | Decision tree, Naïve Bayes, Random Forest, SVM, Adaptive Boosting machine learning models | Adaptive boosting model with Decision Tree (Ada/DT) offers best performance at discriminating between healthy individuals and those with Parkinson's (acc = 0.79, AUC = 0.82). Ada/DT also best performing model to identify differences between healthy individuals, individuals with mild cognitive impairment (MCI), those with Parkinson's and those with Parkinson's and a MCI (acc = 0.85, AUC = 0.96). |
| ***Anomaly Detection*** | | | | | | | | | | |
| ***Title*** | ***Authors*** | ***Year*** | ***Telecare or Telehealth*** | ***Primary or Secondary Data*** | ***Focus of Technology*** | ***Function of Technology*** | ***Technology being used*** | ***Data streams*** | ***Statistical method of analysis*** | ***Study Findings*** |
|  |  |  |  |  |  |  |  |  |  |  |
| Collaborative Multi-Expert Active Learning for Mobile Health Monitoring: Architecture, Algorithms, and Evaluation | Saeedi, R.; Sasani, K.; Gebremedhin, A. H. | 2020 | Telecare | Secondary | Monitoring system | Mobile health monitoring system that features machine learning models capable of adapting to new configurations, contexts or user needs (i.e. missing data) | System capable of incorporating various sensors | Sensor data used in datasets: Accelerometer, gyroscope and magnetometer | Random forest machine learning algorithm with k-means clustering to categorize unlabeled target data | Activity recognition accuracy of over 85% and 92% for each dataset by labelling only 15% of unlabeled data |
| Remote Patient Monitoring Using Mobile Health for Total Knee Arthroplasty: Validation of a Wearable and Machine Learning-Based Surveillance Platform | Ramkumar, P. N.; Haeberle, H. S.; Ramanathan, D.; Cantrell, W. A.; Navarro, S. M.; Mont, M. A.; Bloomfield, M.; Patterson, B. M. | 2019 | Telehealth | Primary | Knee arthroplasty | Remote patient monitoring system for TKA | Neoprene knee sleeve and smartphone | Mobility data recorded through smartphone accelerometer, gyroscope and magnetometer. Weekly knee range of motion (RoM) obtained from sleeve. Smartphone used to collect PROMs, opiod consumption and compliance with exercise programme | Motion-based machine learning software development kit | Remotely acquired continuous data from patients undergoing TKA capable of evaluating patient's progression in terms of mobility and rehabiliation |
| Mining Productive-Associated Periodic-Frequent Patterns in Body Sensor Data for Smart Home Care | Ismail, W. N.; Hassan, M. M. | 2017 | Telecare | Secondary | Monitoring system | A system to pattern mine productive-associated periodic-frequent patterns from home sensors to promote important decision making | Various personal sensors (unspecified) | Physiological data from sensors recorded continuously (heart rate, respiratory rate, blood O2 saturation, diastolic blood pressure and body temperature) | PPFP-growth (Productive Periodic-Frequent Pattern) algorithm | Model offers significant reduction in runtime and efficiency compared to other similar models. PFPP-growth up to 4 times faster than others depending on parameters |
| Closing the loop from continuous M-health monitoring to fuzzy logic-based optimized recommendations | Benharref, A.; Serhani, M. A.; Nujum, A. R. | 2014 | Telecare | Primary | Monitoring system | To provide optimised recommendations to physicians about intervention requirements for patients | Zephyr BioHarness-3 and iBGStar Blood Glucose Meter. Mobile device: iPhone 4S | Blood pressure, heart rate and blood sugar (additional data input including lab tests and historical data) | Fuzzy rules-based expert system | Fuzzy expert system with intelligent monitoring and analytics techniques provided a high accuracy of collected data and valid advice |
| Intelligent remote health monitoring using evident-based DSS for automated assistance | Serhani, M. A.; Benharref, A.; Nujum, A. R. | 2014 | Telehealth | Secondary | Monitoring system | Decision support system offer assistance to an individual based on vitals | Zephyr HxM Heart Rate and BioHarness 3 | Extracted ECG features: Example QRS duration, RR interval, QT and ST segments | Incremental learning machine learning models: Neural network, Decision tree, AutoMLP, W-OneR, Naïve Bayes, RuleModel and J48graft | Best performing model on HR inputs only = RuleModel which detected 7 out of 12 classes correctly (accuracy ~ 60%). Study with HR and ECG inputs: best model again RuleModel which now offers classification accuracy of 90% |
| A double closed loop to enhance the quality of life of Parkinson's Disease patients: REMPARK system | Sama, A.; Perez-Lopez, C.; Rodriguez-Martin, D.; Moreno-Arostegui, J. M.; Rovira, J.; Ahlrichs, C.; Castro, R.; Cevada, J.; Graca, R.; Guimaraes, V.; Pina, B.; Counihan, T.; Lewy, H.; Annicchiarico, R.; Bayes, A.; Rodriguez-Molinero, A.; Cabestany, J. | 2014 | Telehealth | Primary | Parkinson's | To identify undesirable changes in walking patterns and provide help to remedy this | REMPARK Movement sensor | triaxial accelerometer, gyroscope and magnetometer | SVM model | ML model not been validated for accuracy |
| PERFORM: a system for monitoring, assessment and management of patients with Parkinson's disease | Tzallas, A. T.; Tsipouras, M. G.; Rigas, G.; Tsalikakis, D. G.; Karvounis, E. C.; Chondrogiorgi, M.; Psomadellis, F.; Cancela, J.; Pastorino, M.; Waldmeyer, M. T.; Konitsiotis, S.; Fotiadis, D. I. | 2014 | Telehealth | Primary | Parkinson's | Continuous remote monitoring system capable of evaluating Parkinson's motor symptoms | PERFORM wearable multi-sensor monitor unit: Four tri-axial accelerometers and one accelerometer/gyroscope on the waist | Extracted features: Tremor - time and frequency domain characteristics (including features indicative of low frequency movements). Spatial features based on gravity forces applied to accelerometers. LID - mean signal value, sd, entropy, energy etc. Bradykinesia - approx entropy, sample entropy, RMS, cross correlation and range. FoG - entropy of the signal of each sensor | Tremor: Two Hidden Markov models, Levodopa-induced dyskinesia (LID): C4.5 decision tree, Bradykinesia: SVM, FoG: random forest | Tremor: 87% classification accuracy (mean absolute error = 0.088), LID: 85.4% accuracy (mae = 0.31), bradykinesia: 74.5% accuracy (mae = 0.25), FoG: 79% accuracy (mae = 0.79) |
| An automatic rules extraction approach to support OSA events detection in an mHealth system | Sannino, G.; De Falco, I.; De Pietro, G. | 2014 | Telehealth | Secondary | Sleep apnea | To detect the presence of OSA | ECG sensor | Single channel ECG data. Factors extracted from ECG via GHRV: 6 from frequency domain, 4 from time domain and 2 non-linear parameters | Differential Evolution based DEREx system | DEREx rules-based method compared with machine learning classifiers (AdaBoost, Bagging, Random Forest, Logistic Regression and SVM) for OSA classification. DEREx has joint highest accuracy (88.57%) and clear highest results in sensitivity (90.32%) and specificity (90.25%) |
| A randomized controlled trial comparing health and quality of life of lung transplant recipients following nurse and computer-based triage utilizing home spirometry monitoring | Finkelstein, S. M.; Lindgren, B. R.; Robiner, W.; Lindquist, R.; Hertz, M.; Carlin, B. P.; VanWormer, A. | 2013 | Telehealth | Primary | Lung transplant | To provide automatically generated triage decisions | Spirometer | forced vital capacity (FEV1), mid-flow and peak flow plus daily symptom questionaire | Bayesian Decision Support Algorithm | RCT shows no significant difference in physical or quality of life measures between intervention and control, indicating algorithm-based triage is at least as good as nurse-based triage |
| An awareness approach to analyze ECG streaming data | Don, S.; Chung, D.; Choi, E.; Min, D. | 2013 | Telehealth | Secondary | Monitoring system | To remotely monitor patients and make decision on intervention required | ECG data | ECG feature extraction: PR segment, ST segment, beats, QRS interval, PR interval, QT interval and RR interval, wave duration and amplitude also included | Rules-based CEP with ontological awareness model | Experimental results indicate that this model significantly reduces the decision-making workload of medical analysts |
| Dynamic self-adaptive remote health monitoring system for diabetics | Suh, M. K.; Moin, T.; Woodbridge, J.; Lan, M.; Ghasemzadeh, H.; Bui, A.; Ahmadi, S.; Sarrafzadeh, M. | 2012 | Telehealth | Primary | Diabetes | Monitoring architecture designed reduce the number of daily tasks (related to the monitoring of their condition) required to be performed by diabetes patients | Blood glucose sensor | 1688 data points total: Three daily measurements of blood sugar and answer four questions about health a day | Apriori Association Rule Learning algorithm | Developed algorithm capable of reducing the number of tasks required of a patient by up to 28.6% with minimum support of 0.95, minimum confidence of 0.97 and maximum time window of 2 days |
| Heart failure analysis dashboard for patient's remote monitoring combining multiple artificial intelligence technologies | Guidi, G.; Pettenati, M. C.; Miniati, R.; Iadanza, E. | 2012 | Telehealth | Secondary | Heart failure | Computer Decision Support System (CDSS) to assess the current severity of individuals' heart failure | "Device for automatic detection of patient's vital signs at home or other point of care" I.e unspecified | Age, sex, weight, systolic and diastolic blood pressure, heart rate, New York Heart Association classification, ejection fraction, brain natriuretic peptide and ECG parameters (atrial fibrillation, left bundle branch block and ventricular tachycardia) | PCS block relies on the following ML models: Neural Network, SVM, Classification tree (CART) and Fuzzy Expert System | CART model offered best performance with a test and training accuracy of 77.8% and 84% respectively |
| Wearable Cuff-Less Blood Pressure Estimation at Home via Pulse Transit Time | Ganti, V. G.; Carek, A. M.; Nevius, B. N.; Heller, J. A.; Etemadi, M.; Inan, O. T. | 2021 | Telehealth | Primary | Blood pressure | To estimate blood pressure in a non-invasive cuff-less manner | SeismoWatch 2.0 | Single-lead electrocardiogram, tri-axial seismocardiogram and multi-wavelength photoplethysmogram, accelerometer, gyroscope, temperature sensor, pressure sensor and humidity sensor | Multiple linear regression | Correlation coefficient = 0.69 and a RMSE of 2.72 mmHg |
| A pilot study towards a smart-health framework to collect and analyze biomarkers with low-cost and flexible wearables | Rahman, M. J.; Morshed, B. I.; Harmon, B.; Rahman, M. | 2022 | Telecare | Primary | Monitoring system | Identify disease-related events of interest | Smartphone and personal sensors | Body temperature (temp sensor), oxygen saturation (finger pulse oximeter), heart rate (smart wristband) in combination with self-reported symptoms | Random forest machine learning algorithm | Heart rate variability measures are within normative values (95% confidence). System enables automatic community-wide monitoring of symptom severity in tandem with personal monitoring. System leads to reduction of storage requirements in the order of 1,000 |
| Design of a real-time and Continua-based framework for Care Guideline Recommendations | Lin, Y. F.; Shie, H. H.; Yang, Y. C.; Tseng, V. S. | 2014 | Telehealth | Primary and secondary | Monitoring system | To monitor and predict vital signs of patients (including anomalous conditions) to provide caregivers with recommendations | Electrocardiograph, pulse oximeter, capnograph, noninvasive arterial blood pressure monitor, peak flow meter and pressure monitor in secondary data collection source | Pulse oximetry, heart rate and arterial blood pressure | Position Pairs Set algorithm used for data mining | Predictive model has recall ≥ 0.55 and F-measure ~ 0.6 |
| An IoT-based framework for remote fall monitoring | Al-Kababji, A.; Amira, A.; Bensaali, F.; Jarouf, A.; Shidqi, L.; Djelouat, H. | 2021 | Telecare | Secondary | Fall monitoring | To detect if a fall has occurred | Accelerometer and ECG sensor | Triaxial acclereometer data and ECG. Features extracted from accelerometer and ECG include CWT, SVM and SMA | KNN, ENN, BDT and VM machine learning models | ENN model offered best performance as VM results dictated by results of KNN and ENN. ENN model accuracy = 99.15% and F1 = 98.81% |
| Automated smart home assessment to support pain management: multiple methods analysis | Fritz, R. L.; Wilson, M.; Dermody, G.; Schmitter-Edgecombe, M.; Cook, D. J. | 2020 | Telecare | Primary | Pain management | To detect pain-related behaviours to allow for an automated assessment and intervention | Passive infrared motion sensor, magnetic door use, light, temperature and humidity | Only passive infrared motion and door use featured in analysis. Behavioural markers extracted include mean, median, sd, mex, min, zero crossings, mean crossings, iqr, skewness, kurtosis and signal energy | Random forest machine learning algorithm | RF ML model classification accuracy = 0.7, sensitivity = 0.72 and specificity = 0.69. Standard anomaly detection method not augmented by qualitative work achieved accuracy of 0.16 |
| The SmartHabits: An Intelligent Privacy-Aware Home Care Assistance System | Grguric, A.; Mosmondor, M.; Huljenic, D. | 2019 | Telecare | Primary | Monitoring system | An intelligent home care assistance system, capable of identifying anomalies | Various sensors employed in home: Door/window. Wall plug, motion, luminance, temperature and humidity | Sensor data (unspecified) | Rule Recommendation Engine utilises k-means clustering to process data for anomaly detection | System learned an average of 23 patterns per household in the first 30 days. System offers high accuracy for anomaly detection. 61% of proposed rules were accepted by caregivers. |
| Automated remote fall detection using impact features from video and audio | Geertsema, E. E.; Visser, G. H.; Viergever, M. A.; Kalitzin, S. N. | 2019 | Telecare | Secondary | Fall monitoring | To allow for the remote detection of high impact falls | Video and audio recordings (no further specification) | EXTRACTED FEATURES - Video: Maximum downward acceleration, deceleration, point of maximum vertical downward velocity. Audio: Sound peak amplitude | SVM with a radial basis function kernel | Performed classification on Le2i database produced 90% sensitivity for the detection of falls with a specificity of 92% |
| Cloud-Based Behavioral Monitoring in Smart Homes | Mora, N.; Matrella, G.; Ciampolini, P. | 2018 | Telecare | Primary | Monitoring system | Monitoring architecture to detect behavioural change | Various environmental sensors | Sensors: Motion detection, magnetic contact, bed occupancy, chair occupancy, toilet presence, fridge and power meter | Real-valued regression and discrete-valued regression depending on nature of sensor. Additionally, sensor profile comparison and multivariate habits clustering | Concept proven, system able to extract expressive indications from indirect, continuous monitoring fully automatically. |
| Lynx: Automatic Elderly Behavior Prediction in Home Telecare | Lopez-Guede, J. M.; Moreno-Fernandez-De-Leceta, A.; Martinez-Garcia, A.; Grana, M. | 2015 | Telecare | Primary | Monitoring system | To identify behaviour of individuals and alert care providers to any abnormal behaviour | Various sensors employed (environmental, physiological and embedded service and audiovisual sensors), plug and play so can be changed | Physiological: e.g. Temperature, heart pulse, blood pressure etc. Environmental: e.g. smoke detector, presence, open doors, temperature, humidity etc | Ontology-based Decision support system with decision tree ML model | With temporal sliding window method: overall accuracy of system with cross-validation = 81.8% |
| ***Prediction*** | | | | | | | | | | |
| ***Title*** | ***Authors*** | ***Year*** | ***Telecare or Telehealth*** | ***Primary or Secondary Data*** | ***Focus of Technology*** | ***Function of Technology*** | ***Technology being used*** | ***Data streams*** | ***Statistical method of analysis*** | ***Study Findings*** |
|  |  |  |  |  |  |  |  |  |  |  |
| Detecting asthma exacerbations using daily home monitoring and machine learning | Zhang, O.; Minku, L. L.; Gonem, S. | 2020 | Telehealth | Secondary | Asthma | To detect asthma exacerbations from daily self-monitoring of peak expiratory flow and asthma symptoms | Peak expiratory flow meter and symptom diary | Morning, evening and mean peak expiratory flow rates, morning and evening symptoms, number of puffs of reliever inhaler used during overnight and daytime periods, total of morning and evening symptom scores and overnight and daytime reliever inhaler usage. Also binary classifier for whether a person woke up during the night | Multiple ML models considered: logistic regression, naïve bayes, decision trees and perceptron | Logistic regression with principal component analysis for variable selection offered best performance: sensitivity = 90%, specificity = 83% for asthma exacerbations. AUC = 85% |
| Predicting post-discharge cancer surgery complications via telemonitoring of patient-reported outcomes and patient-generated health data | Rossi, L. A.; Melstrom, L. G.; Fong, Y.; Sun, V. | 2021 | Telehealth | Secondary | Cancer | To predict post-discharge cancer surgery complications from telemonitored data | VivoFit wristband pedometers and ePROs to assess symptoms | From VivoFit: daily steps to extract - max, min, median counts, s.d., slope and intercept of linear interpolation, differences with respect to baseline, fraction of days patient wore pedometer. From ePROs: answers with scores ranging 0-10 for assessed discomfort | Logistic regression modelling via nested cross-validation with L1 or L2 regularisation | Best performing model, using data from ePROs and sensor recordings - with 25 features, offers an AUROC of 0.74 |
| Unsupervised Assessment of Balance and Falls Risk Using a Smartphone and Machine Learning | Greene, B. R.; McManus, K.; Ader, L. G. M.; Caulfield, B. | 2021 | Telecare | Primary | Fall monitoring | To assess balance and risk of a fall | Smartphone | Questionnaire answers focussing on demographic data and risk factors. Inertial sensor features: RMS acceleration total and on each axis, RMS angular velocity average and on each axis, median frequency acceleration, spectral edge frequency acceleration, spectral entropy acceleration, median frequency angular velocity, spectral edge frequency angular velocity, spectral entropy angular velocity, age, height and weight | Fall risk estimate calculated through fusion ML model combining logistic regression models analysing both the questionnaire answers and sensor data | Ternary classification: Accuracy of falls classification = 61.78%, class sensitivity of 86.9% (non-faller), 25% (1 fall) and 8.96% (recurrent faller). Binary classification: Accuracy of falls classification = 69.87%, class sensitivty of 86.9% (non-faller) and 37.9% (faller) |
| Predicting Outcomes in Patients Undergoing Pancreatectomy Using Wearable Technology and Machine Learning: Prospective Cohort Study | Cos, H.; Li, D.; Williams, G.; Chininis, J.; Dai, R.; Zhang, J.; Srivastava, R.; Raper, L.; Sanford, D.; Hawkins, W.; Lu, C.; Hammill, C. W. | 2021 | Telehealth | Primary | Pancreatectomy | To predict patient outcomes after pancreatectomy through data obtained from wearable sensors | Fitbit Inspire HR | Three types of features extracted from data: statistical, semantic and biobehavioural rhythmic features. Statistical: first and second order features extracted from daily step count, heart rate and sleep time-series data. Semantic: e.g. time in bed, minutes to fall asleep, daily sedentary time etc. Biobehavioural: e.g. stability, variability, mean of last 5 hours, relative amplitude, phase etc | Multiple ML models considered: random forest, gradient boosted trees, kNN, SVM with linear kernel and logistic regression with L1 penalty | All ML models outperformed the standard ACS-NSQIP surgical risk calculator which had an area under the receiving operating characteristic (AUROC) of 0.6333. The best performing model was a gradient boosted trees model trained on patient clinical characteristics and patient activity which offered AUROC of 0.7875 |
| A nurse-driven method for developing artificial intelligence in "smart" homes for aging-in-place | Fritz, R. L.; Dermody, G. | 2019 | Telecare | Primary | Monitoring system | To identify and/or predict a change in the health state of an indiviudal to allow for intervention | Environmental sensors | Five environmental sensors (infrared motion, contact, light, temperature and humidity) coupled with regular telehealth visits | Machine learning model operating with both qualitative and quantitative inputs | Framework with no study findings (not tested with data in paper) |
| OnTrack: development and feasibility of a smartphone app designed to predict and prevent dietary lapses | Forman, E. M.; Goldstein, S. P.; Zhang, F.; Evans, B. C.; Manasse, S. M.; Butryn, M. L.; Juarascio, A. S.; Abichandani, P.; Martin, G. J.; Foster, G. D. | 2019 | Telecare | Primary | Weight | Smartphone app (OnTrack) to predict dietary lapses | Semi-random surveys implemented in app multiple times a day | Answer to survey questions: generally binary yes:no answers (cravings, hunger, tiredness, temptations, alochol consumption, exercise etc). Continuous hours of sleep recorded and 5 point scale answers to questions focussing on affect, self-efficacy, motivation and cognitive load | Ensemble model utilizing logit boost, bagging, random subspace, random forest and Bayes net | Algorithm able to achieve an accuracy of 72%, sensitivity of 70% and specificity of 72%. Negative predictive value of 80% achieved |
| Early anomaly detection in smart home: A causal association rule-based approach | Hela, S.; Amel, B.; Badran, R. | 2018 | Telecare | Secondary | Monitoring system | Early anomaly detection from sensor data | on/off sensors, door state sensors, bed occupancy sensors, temperature sensors etc | States of each sensor - UNCLEAR EXACTLY WHAT DATA STREAMS USED | Markov Logic Network ML model | Precision approaches 100% precision at 3-4 lead in window. Correctness peaks at 4 minutes at around 95%. Recall = 90% Performance generally peaks between 3 and 4 minutes |
| Improving Prediction of Risk of Hospital Admission in Chronic Obstructive Pulmonary Disease: Application of Machine Learning to Telemonitoring Data | Orchard, P.; Agakova, A.; Pinnock, H.; Burton, C. D.; Sarran, C.; Agakov, F.; McKinstry, B. | 2018 | Telehealth | Secondary | COPD | To assess an individual's risk of hospitalisation due to COPD | Spirometer and oxygen saturation monitor | Daily symptoms(2 = major symptoms, 1 = minor symptoms), pulse and oxygen saturation, spirometry data, antibiotic and corticosteroid use, demographics, BMI, Dyspnoea Scale, St George's Respiratory Questionnaire, previous hospital admissions and comorbidities. Met Office data: season, humidity, temp, air quality and influenza rates etc | Nonparamteric prediction method (sparse maximum-margin classifiers), regularised classifiers based on adaptive extensions of elastic nets, ensembles of boosted classifiers and Long short-term memory multitask neural network | Multitask neural network (fitted with 135 variables) provides best prediction of upcoming hospital admission (AUC = 0.74, 95% CI = 0.673, 0.803). Addition of weather data had no effect on model. Best model for corticosteroid need: AUC = 0.765 (95% CI = 0.738, 0.791) |
| Development of a Computer-Aided Dosage and Telemonitoring System for Patients Under Oral Anticoagulation Therapy | Krumm, H.; Reiss, N.; Burkert, M.; Schmidt, T.; Biehs, S.; Bohr, C.; Gurtler, F.; Horn, H.; Kreutzer, P.; Mewes, P.; Miller, H.; Riest, C.; Romer, C.; Seebold, A.; Sprung, G.; Ziegler, O. | 2018 | Telehealth | Primary | Oral anticoagulation | To allow individuals receiving anticoagulation medication to independently manage their dosage through predictive algorithms | App for reporting international normalised ratio values (INR - important anticoagulation drug measure) | 277 pairs of reported INR values and corresponding doses administered by patient used during training. Technology aims to replace independent doses administered and automatically recommend dosage | Neural network and Model Predictive Control ML techniques | Between 50 and 60% of recommendations satisfied the criteria established |
| Machine learning approaches to personalize early prediction of asthma exacerbations | Finkelstein, J.; Jeong, I. C. | 2016 | Telehealth | Secondary | Asthma | To predict the onset of an asthma exacerbation | Asthma diary and peak expiratory flow meter | PEF data and 20 answers to asthma diary (symptoms present, limitation of activity, self-estimate of asthma, use of inhaler, asthma effect on sleep etc) | Adaptive Bayesian Network, Naïve Bayes classifier and SVM ML models | Using a 7 day window: naïve Bayesian classifier, adaptive bayesian network and SVM were able to predict asthma exacerbation on day 8 with: (sensitivity = 0.8, 1 and 0.84 respectively) (specificity = 0.77, 1 and 0.8 respectively) (accuracy = 0.77, 1 and 0.8 respectively) |
| Remote Health Monitoring Outcome Success Prediction Using Baseline and First Month Intervention Data | Alshurafa, N.; Sideris, C.; Pourhomayoun, M.; Kalantarian, H.; Sarrafzadeh, M.; Eastwood, J. A. | 2017 | Telehealth | Primary | Monitoring system | To predict outcome success of remote health monitoring course after baseline contextual features collected and after one month of data collection | Blood pressure monitor and smartphone | Physical activity (e.g total activity level and time in low, medium and high activity), blood pressure (diastolic and systolic pressures), 12 daily questionnaire responses, 12 weekly questionnaire responses. Features extracted include: extremes, averages, quartiles, moments and peaks | ML models: logistic regression, C4.5 decision trees, k-NN and Naïve bayes | Optimal results using just baseline achieved by k-NN: accuracy = 75.7%, F-measure = 75.5% and ROC = 75.4%. Optimal results using both context and statistical features achieved by logistic regression: accuracy = 91.9%, F-measure = 91.9% and AUC = 95.9% |
| Prediction of exacerbation onset in chronic obstructive pulmonary disease patients | Riis, H. C.; Jensen, M. H.; Cichosz, S. L.; Hejlesen, O. K. | 2016 | Telehealth | Secondary | COPD | To predict the risk of exacerbation in patients with COPD | Telehealth Monitor RTX 3371 (Tunstall Healthcare). Sphygmomanometer and pulse oximeter | Systolic and diastolic blood pressure, mean arterial pressure, pulse, oximeter pulse, oxygen saturation. Features extracted: mean, linear regression, skewness, kurtosis and s.d. | k-NN classification | Best performing classifier achieved: Sensitivity = 73%, specificity = 74%, PPV = 69%, NPV = 78%, accuracy = 74% |
| Predicting the risk of exacerbation in patients with chronic obstructive pulmonary disease using home telehealth measurement data | Mohktar, M. S.; Redmond, S. J.; Antoniades, N. C.; Rochford, P. D.; Pretto, J. J.; Basilakis, J.; Lovell, N. H.; McDonald, C. F. | 2015 | Telehealth | Secondary | COPD | To predict the risk of exacerbation in patients with COPD | TMC-Home device for physiological measurements and questionnaires | Features (distribution mean and s.d., % change of parameter from mean, z-score) extracted from: FEV1, SpO2, respiratory rate, heart rate, temperature and weight | Classification and Regression Tree (CART) algorithm | CART model performance: accuracy = 71.8%, specificity = 80.4% and sensitivity = 61.1%. Most predictive features: FEV1, SpO2 distribution mean, weight distribution mean and weight standard deviation |
| Automatic prediction of chronic obstructive pulmonary disease exacerbations through home telemonitoring of symptoms | Fernandez-Granero, M. A.; Sanchez-Morillo, D.; Leon-Jimenez, A.; Crespo, L. F. | 2014 | Telehealth | Primary | COPD | To allow for the automatic prediction of a COPD exacerbation | Patients given dedicated mobile device for use in this project | Daily questionnaire Automated Questionnaire for the early detection of AECOPD (AQCE) completed. 12 predictor variables obtained from this. 4 additional variables: 3-day moving average of scores and average scores for symptoms associated with major, minor and complimentary symptoms | A probabilistic neural network (PNN) | Out of 41 episodes in the experiment the PNN model was able to detect 33 exacerbations in advance of symptoms appearing (average of 4.8 days prior to onset). Accuracy of 88.3%, sensitivity of 80.5%, specificity = 94.34%. 3 false positives identified |
| A decision support system for the treatment of patients with ventricular assist device support | Karvounis, E. C.; Tsipouras, M. G.; Tzallas, A. T.; Katertsidis, N. S.; Stefanou, K.; Goletsis, Y.; Frigerio, M.; Verde, A.; Caruso, R.; Meyns, B.; Terrovitis, J.; Trivella, M. G.; Fotiadis, D. I. | 2014 | Telehealth | Secondary | Heart failure | To manage and provide remote treatment for patients with Heart Failure, including risk prediction | Ventricular Assist Device (VAD), implantable devices, wearable sensors | Age, INTERMACS profile, platelets, hemoglobin, hematocrit, white blood cells, right atrial pressure, pulmonary artery pressure (max, min, mean), Pulmonary Capillary Wedge, Cardiac index, Reversible pH, heart rate, PA, International Normalised Ratio, Bilirubine, Creatinine, Urea, Na+, Model for end stage liver disease (MELD), MELD UNOS, MELD U+Age, Aspartate Anotransferase and Inotropes | Machine Learning Algorithms: Naïve Bayes, kNN, decision tree, random forest, multilayer perceptron, neural networks and SVM | Decision tree model chosen as good permofrmance and preferred by medical experts due to interpretability of results. Classification accuracy for treatment support tool = 84%, for monitoring tool = 99% |
| An information and communication technology system to detect hypoglycemia in people with type 1 diabetes | Jensen, M. H.; Christensen, T. F.; Tarnow, L.; Johansen, M. D.; Hejlesen, O. K. | 2013 | Telehealth | Primary | Diabetes | To monitor glucose of patient and detect upcoming hypoglycemia | Guardian RT-CGM | Plasma glucose readings in combination with patient-input data such as carbohydrate content of meals | SVM model | SVM model identified 17/17 hypoglycemic events compared to 12/17 for just glucose monitoring. Lead time for hypoglycemic event increased from averge of 0 minutes to 14 minutes. One false alert during total sampling period |
| Predicting changes in glycemic control among adults with prediabetes from activity patterns collected by wearable devices | Patel, M. S.; Polsky, D.; Small, D. S.; Park, S. H.; Evans, C. N.; Harrington, T.; Djaraher, R.; Changolkar, S.; Snider, C. K.; Volpp, K. G. | 2021 | Telehealth | Primary | Diabetes | To predict changes in glycemic control from wearable sensors | Digital weight scale. Half received waist-worn Fitbit Zip and half received wrist-worn Fitbit Charge 2 HR | Demographic data (e.g. age, gender, race, education, marital status and annual income) and patient info (e.g. smoking status, relative with diabetes and taking medication to control blood sugar). Sensor data: measures of physical activity, heart rate data and sleep data | 6 different modelling techniques, 3 regression based: ordinary regression without regularisation, ridge regression and lasso regression. Two tree based: random forest and gradient boosting trees and ensemble incorporating ridge regression, rf and gradient boosting | Ensemble method performs significantly better than all other models in terms of prediction of continuous change in hemoglobin, worsening and improving hemoglobin |
| Wearable sensor derived decompensation index for continuous remote monitoring of COVID-19 diagnosed patients | Richards, D. M.; Tweardy, M. K. J.; Steinhubl, S. R.; Chestek, D. W.; Hoek, T. L. V.; Larimer, K. A.; Wegerich, S. W. | 2021 | Telehealth | Primary | Covid-19 | To predict Covid-19 decompensation event ikelihood | VitalConnect VitalPatch chest patch biosensor | 361 features extracted from: statistical (102), filtered statistical (240), weighted average (4), interaction (8), delta interaction (4), sleep (2) and data quality (1) | Gradient boosted decision trees | Overall AUC of the ROC curve = 0.84 on 308 negative events and 22 positive events |
| Continuous wearable monitoring analytics predict heart failure hospitalization: The link-hf multicenter study | Stehlik, J.; Schmalfuss, C.; Bozkurt, B.; Nativi-Nicolau, J.; Wohlfahrt, P.; Wegerich, S.; Rose, K.; Ray, R.; Schofield, R.; Deswal, A.; Sekaric, J.; Anand, S.; Richards, D.; Hanson, H.; Pipke, M.; Pham, M. | 2020 | Telehealth | Primary | Heart failure | To allow for the prediction of rehospitalisation with heart failure | VitalConnect wearable sensor | ECG waveforms, 3-axis accelerometry, skin impedance, skin temperature and info on activity and posture. Extracted features include: heart rate, HRV, arrhythmia burden, respiratory rate, gross activity, walking, sleep, body tilt and posture | Similarity-based ML modelling (SBM) | Model capable of predicting risk of HF hospitalisation with sensitivity and specificity of 76% and 84.8% for 10-day positive window method. For event-specific window, sensitivity = 87.5% with specificity = 86% |
| Atrial fibrillation burden signature and near-term prediction of stroke: A machine learning analysis | Han, L.; Askari, M.; Altman, R. B.; Schmitt, S. K.; Fan, J.; Bentley, J. P.; Narayan, S. M.; Turakhia, M. P. | 2019 | Telehealth | Secondary | Atrial fibrillation | To predict stroke risk from daily AF burden | Pacemakers and Implantable Cardioverter Defibrillators. Wearable sensors | 30 days worth of information on AF burden used as features in machine learning model | Random forest, Convolutional Neural Network and L1 Regularised Logistic Regression (LASSO) | All machine learning outperformed CHA2DS2-VASc (score for Atrial Fibrillation Stroke Risk). Best performing model was ensemble model encorporating ML with CHA2DS2-VASc. |
| Population exacerbation incidence contains predictive information of acute exacerbations in patients with chronic obstructive pulmonary disease in telecare | Kronborg, T.; Mark, L.; Cichosz, S. L.; Secher, P. H.; Hejlesen, O. | 2018 | Telehealth | Secondary | COPD | To investigate if population exacerbation incidence of COPD can be used to predict acute exacerbation | Pulse oximeter, blood pressure monitor and a scale. Questionnaire completed with questions related to symptoms | Oxygen saturation, pulse, weight, blood pressure and questionnaire answers | Logistic regression based ML model | Optimal predictor combination with exclusion of patient and population incidence of exacerbation: AUC = 0.627, s.d. = 0.108. Optimal predictor combination with inclusion of patient and population exacerbation: AUC = 0.746, s.d. = 0.05 |
| Cloud-Based Smart Health Monitoring System for Automatic Cardiovascular and Fall Risk Assessment in Hypertensive Patients | Melillo, P.; Orrico, A.; Scala, P.; Crispino, F.; Pecchia, L. | 2015 | Telehealth | Secondary | Cardiovascular disease and fall monitoring | A platform to collect and automatically analyse biomedical signals to provide a risk assessment for vascular events and falls in hypertensive patients | Zephyr BioHarness-3 | ECG data extracted features: time-domain, frequency-domain and nonlinear. Activity and sleep parameters measured through actigraphy | Naïve Bayes, decision trees, random forest, rotation forest, Adaboost M1 and RUSBoost, SVM, artificial neural network | STUDY DOES NOT MAKE CLEAR WHICH MODEL PERFORMED BEST. Best algorithm for cardiovascular risk: accuracy = 83.9%, sensitivity = 71.4%, specificity = 85.7%. Best algorithm for falls risk: accuracy = 72%, sensitivity = 51.1%, specificity = 80.2% |
| ***Activity Recognition*** | | | | | | | | | | |
| ***Title*** | ***Authors*** | ***Year*** | ***Telecare or Telehealth*** | ***Primary or Secondary Data*** | ***Focus of Technology*** | ***Function of Technology*** | ***Technology being used*** | ***Data streams*** | ***Statistical method of analysis*** | ***Study Findings*** |
|  |  |  |  |  |  |  |  |  |  |  |
| D-SORM: A digital solution for remote monitoring based on the attitude of wearable devices | Abbas, M.; Somme, D.; Le Bouquin Jeannes, R. | 2021 | Telehealth | Secondary | Activity recognition | To provide arm tele-rehabiliation and human motion tracking for elderly care (focusing on motion tracking) | MARG (Magnetic, Angular Rate, Gravity) sensor | Tri-axial accelerometer, gyroscope and magnetometer data | Feature fusion based ML model (kNN, RF, DNN, Random Subspace, CNN and Long Short-Term Memory) | Best performing model was combination of CNN, dense layers and LSTM and provided an accuracy of 97.7% trained on both raw data and extracted features |
| Highly Accurate Bathroom Activity Recognition Using Infrared Proximity Sensors | Chapron, K.; Lapointe, P.; Bouchard, K.; Gaboury, S. | 2020 | Telecare | Primary | Activity recognition | To facilitate the identification of activities performed in the bathroom | Raspberry Pi Zero W based motion sensors behind toilet and shower | Shower sensor extracts ϒ (average value of sensor, distance from sensor) and standard deviation of ϒ. Toilet sensor identifies 4 actions: ϱ1 = sitting on toilet, ϱ2 = sitting and leaning on toilet, ϱ3 = the act of standing close to the toilet and ϱ4 = the act of standing far from the toilet | Algorithm for classification of activity being performed (not ML) | Recall most important metric since it involves activities that actually happened but have not be recognised. Toilet recall = 0.9791 and shower recall = 1.0 |
| Recognition of Sedentary Behavior by Machine Learning Analysis of Wearable Sensors during Activities of Daily Living for Telemedical Assessment of Cardiovascular Risk | Kantoch, E. | 2018 | Telehealth | Primary | Activity recognition | To identify sedentary behaviour from wearable sensors | Prototype smart shirt | Accelerometer, ambient light sensor, temperature sensor, optical heart rate sensor | ML models: LDA, SVM, k-NN, binary decision trees, naïve bayes and artificial neural network (ANN) | Best performing models: SVM, binary decision trees and ANN. Accuracy = 96.67%, sensitivity = 93.33% and specificity = 97.78% |
| Activity Recognition for Persons With Stroke Using Mobile Phone Technology: Toward Improved Performance in a Home Setting | O'Brien, M. K.; Shawen, N.; Mummidisetty, C. K.; Kaur, S.; Bo, X.; Poellabauer, C.; Kording, K.; Jayaraman, A. | 2017 | Telecare | Primary | Activity recognition | To identify activities being performed by individuals | Samsung Galaxy S4 | Accelerometer and gyroscope data (including mean, range, IQR, moments, histogram, moments of derivative, mean of squared norm, sum of axial sds, pearson correlation coefficient, mean cross products, absolute mean of cross products, power spectra, mean power). Barometer data (moments of derivative, SD, range, IQR, slope of linear regression) | Random forest machine learning algorithm | Activity recognition models for use with stroke patients that are trained with data from healthy individuals only perform significantly worse (53%) than models trained with data from other stroke victims (75%) |
| Human Activity Recognition from Smart-Phone Sensor Data using a Multi-Class Ensemble Learning in Home Monitoring | Ghose, S.; Mitra, J.; Karunanithi, M.; Dowling, J. | 2015 | Telecare | Secondary | Activity recognition | To identify the activity being completed by system user | Samsung Galaxy S2 smartphone | Triaxial accelerometer and gyroscope for linear accelerations and angular velocity | Random forest ML algorithm | Model achieved near perfect precision and recall for identifying walking, standing, sitting and laying. Slightly lower performance for classification of walking up (recall = 0.97, precision = 0.94) and downstairs (recall = 0.98, precision = 0.95) blamed on not having a sensitive enough gyroscope sensor |
| A Kinect based intelligent e-rehabilitation system in physical therapy | Gal, N.; Andrei, D.; Nemes, D. I.; Nadasan, E.; Stoicu-Tivadar, V. | 2015 | Telehealth | Primary | Activity recognition | To record posture and motion of patient | Microsoft 3D Kinect depth sensor | Angles of 20 joints in human body recorded | Fuzzy, rule-based AI inference system to identify movement | Kinect able to detect posture and motion range of joints |
| Sensor-based activity recognition using extended belief rule-based inference methodology | Calzada, A.; Liu, J.; Nugent, C. D.; Wang, H.; Martinez, L. | 2014 | Telecare | Secondary | Activity recognition | To recognise an individual's activity from sensors | 14 binary ON/OFF sensors | 14 sensors: microwave, hall-toilet door, hall- bathroom door, cups’ cupboard, fridge, plates’ cupboard, front door, dishwasher, toilet flush, freezer, pans’ cupboard, washing machine, groceries’ cupboard and hall-bedroom door | Extended belief rule-based inference methodology (RIMER+) using hamming. Compared to ML models like Naïve Bayes, SVM, k-NN and decision tree | RIMER w/ DRA and hamming outperforms all ML models: mean accuracy 96.59%, with random number of sensors deactivated, RIMER w/ DMA and hamming still outperforms other models: 4 and 5 deactivated - CV10 acc = 97.55% |
| A depth video sensor-based life-logging human activity recognition system for elderly care in smart indoor environments | Jalal, A.; Kamal, S.; Kim, D. | 2014 | Telecare | Primary and secondary | Activity recognition | Identify and classify human activity during task performance | Microsoft 3D Kinect depth sensor | Angles of 15 joints in human body recorded | Hidden Markov ML Model | HMM achieved mean recognition rate of 92.33%, 93.58% and 90.33% for smart home, smart office and smart hospital settings respectively |
| Machine Learning-Based Classification of Human Behaviors and Falls in Restroom via Dual Doppler Radar Measurements | Saho, K.; Hayashi, S.; Tsuyama, M.; Meng, L.; Masugi, M. | 2022 | Telecare | Primary | Activity recognition and fall monitoring | To identify the actions being completed by an individual in the toilet (including falls) | Two doppler radar sensors (dual doppler): one on ceiling and one on wall behind toilet | Short-time Fourier transforms of the received signals calculated to generate spectrogram images for use in modelling. For RF and SVM methods, features of acceleration and jerk were more effective for modelling than the velocity parameters obtained from radar measurements and were included | Convolutional Neural Network, Long Short-Term Memory, SVM and random forest ML algorithms | Best method for classification of behaviour was CNN model (using both sensor data) which gave an accuracy of 95.6%. In addition, this method offered a 100% classification of falls. Wall sensor performed better on its own that ceiling sensor did on its own |
| Utilizing machine learning to recognize human activities for elderly and homecare | Alaraj, R.; Alshammari, R. | 2020 | Telecare | Secondary | Activity recognition | To determine whether ML models can classify the activity of users | Samsung Galaxy S II | Triaxial accelerometer and gyroscope data - 561 features extracted | Ridged regression and deep neural networks | Almost all activities recognised correctly >98% of the time |
| A sequence-to-sequence model-based deep learning approach for recognizing activity of daily living for senior care | Zhu, H.; Chen, H.; Brown, R. | 2018 | Telecare | Secondary | Activity recognition | To allow for the identification and recognition of complex activities of daily living (ADL) | Variety of environmental and motion sensors. All sensors can be integrated into this framework. | Oppo dataset: accelerometer, gyroscope, magnetometer and inertial measurements. CASAS-I: 51 pressure sensors, 21 contact switch sensors and 3 analog sensors. | Gated Recurrent Unit-based Sequence-to-Sequence model with encoder and decoder networks both recurrent neural networks (S2S_GRU with RNN) | In all experiments with datasets: S2S_GRU outperforms S2S w/ Long Short-Term Memory, SVM, Hidden Markov Model and Naïve Bayes. Accuracy = 77.5%, Average precision = 76%, Average recall = 75.8%, Average F1 Score = 75.4%. ABLD significantly lower for this model |
